# Supplementary material for: Randomised controlled feasibility trial of standard wound management versus negative-pressure wound therapy in the treatment of adult patients having surgical incisions for hip fractures
Source: BMJ Open. 2018 Apr 12;8(4):e020632. doi: 10.1136/bmjopen-2017-020632 (PMC5898317; doi:10.1136/bmjopen-2017-020632)
Supplement: Author's manuscript [file bmjopen-2017-020632.draft_revisions.pdf]

BMJ Open is committed to open peer review. As part of this commitment we make the peer review history of every article we publish publicly available.

When an article is published we post the peer reviewers' comments and the authors' responses online. We also post the versions of the paper that were used during peer review. These are the versions that the peer review comments apply to.

The versions of the paper that follow are the versions that were submitted during the peer review process. They are not the versions of record or the final published versions. They should not be cited or distributed as the published version of this manuscript.

BMJ Open is an open access journal and the full, final, typeset and author-corrected version of record of the manuscript is available on our site with no access controls, subscription charges or pay-per-view fees (http://bmjopen.bmj.com).

If you have any questions on BMJ Open's open peer review process please email info.bmjopen@bmj.com

BMJ Open

# WHITE 7 - WHISH – Wound Healing in Surgery for Hip fractures
## A Randomised Controlled Feasibility Trial of standard wound management versus negative pressure wound therapy in the treatment of adult patients having surgical incisions for hip fractures



| | |
|---|---|
| Journal: | *BMJ Open* |
| Manuscript ID | bmjopen-2017-020632 |
| Article Type: | Protocol |
| Date Submitted by the Author: | 15-Nov-2017 |





| | |
|---|---|
| Complete List of Authors: | Masters, James; University of Oxford, Oxford Trauma, Nuffield Department of Orthopaedics, Rheumatology and Musculoskeletal sciences (NDORMS)<br>Achten, Juul; University of Oxford, NDORMS<br>Cook, Jonathan; University of Oxford, NDORMS<br>Dritsaki, Melina; University of Warwick,<br>Sansom, Lucy; University of Oxford, Nuffield Department of Orthopaedics, Rheumatology, and Musculoskeletal Sciences<br>Costa, Matthew; University of Oxford, Nuffield Department of Orthopaedics, Rheumatology, and Musculoskeletal Sciences |





| | |
|---|---|
| Keywords: | Hip < ORTHOPAEDIC & TRAUMA SURGERY, Surgical Site Infection, Negative Pressure Wound Therapy |
| | |



SCHOLARONE™ Manuscripts

For peer review only

# WHITE 7 - WHISH – Wound Healing in Surgery for Hip fractures A Randomised Controlled Feasibility Trial of standard wound management versus negative pressure wound therapy in the treatment of adult patients having surgical incisions for hip fractures.

Corresponding author
James Masters
Oxford Trauma, NDORMS
University of Oxford
Oxford, OX3 9DU
James.masters@ndorms.ox.ac.uk
01865 223114

James Masters[1] Juul Achten[1], Jonathan Cook[2], Melina Dritsaki[3], Lucy Sansom[1] and Matthew L Costa[1].

[1] Oxford Trauma, Nuffield Department of Orthopaedics, Rheumatology & Musculoskeletal Sciences, University of Oxford, Oxford, UK
[2] Centre for Statistics in Medicine, Nuffield Department of Orthopaedics, Rheumatology & Musculoskeletal Sciences, University of Oxford, Oxford, UK
[3] Oxford Clinical Trial Unit, Nuffield Department of Orthopaedics, Rheumatology & Musculoskeletal Sciences, University of Oxford, Oxford, UK

Word Count: 3856 Based on version number 4.0 1st November 2017

For peer review only

**Abstract**

**Introduction**

Deep wound infection is a catastrophic complication after hip fracture surgery. However, current understanding of infection rates in this population is limited. Many technologies such as incisional negative pressure wound therapy (NPWT) show promise in reducing the rate of infection. This trial is a feasibility study looking to establish a precise estimate of the rate of deep infection after hip fracture treatment in patients treated with NPWT versus standard dressing following hip fracture surgery.

**Methods and Analysis**

A randomised controlled trial of 464 patients will be run across multiple centres. It is embedded in the World Hip Trauma Evaluation cohort study. Any patient over the age of 65 years having surgery for hip fracture is eligible unless they are being treated with percutaneous screw fixation. A web based randomisation sequence will stratify patients by centre. Patients will be allocated to either NPWT or standard care on a 1:1 basis. The primary outcome measure is the Centre for Disease Control definition of deep infection at 30 days. Follow up at 4 months will also assess deep infection and the core outcome dataset for hip fractures. This includes health related quality of life (EQ-5D-5L), mobility, mortality and late complications such as further surgery. The primary analysis will be intention to treat.

**Ethics and Dissemination**

Oxford C Research Ethics Committee granted ethical approval on 28/04/2017, 17/SC/0207. This results of this study will be reported in a peer reviewed publication and inform the design of a future full-scale trial.

**Trial registration number**

ISRCTN55305726;

Abstract word Count: 252

For peer review only

Strengths and limitations of the study

- This prospective trial will estimate the true rate of infection according to nationally recognised criteria. .
- Robust data collection systems will be used to determine the rate of deep infection.
- This study will include the full range of hip fracture patients including those without capacity, across multiple centres ensuring good external validity to our findings.
- The potential limitation will be to accurately capture those cases of infection where patients move through different institutions and care pathways.

For peer review only

**Introduction**

Hip fracture is one of the biggest challenges facing patients and healthcare systems at present. Worldwide there are 1.3 million hip fractures with more than 70,000 hip fractures in the UK every year (1). These figures are projected to rise to >100,000 by 2020 in the UK (1) and more than 6 million by 2050 worldwide(2). The global cost of this clinical problem is estimated at 1.75 million disability adjusted life years lost and represents 1.4% of the total healthcare burden in established market economies (3).

The overwhelming majority of hip fractures are treated surgically. The principal objective of surgical treatment is to enable patients to return to weight bearing mobilisation as soon as possible.

As with all surgical treatments, one of the most important potential complications is surgical site infection (SSI). Hip fracture patients are particularly vulnerable to wound problems/surgical site infection. The reasons for this vulnerability are multifactorial. For instance, sixty percent of hip fracture patients have at least one major medical co-morbidity (4) such as steroid or other immunosuppressive medication use, malignancy and diabetes. These co-morbidities sit alongside age related deterioration of immune function.

Surgical site infection can be either superficial or deep. Superficial infections only involve the skin and subcutaneous tissue, whereas 'deep' infections involve the deeper layers and the metalwork implanted in the hip. Deep infection has profound consequences for hip fracture patients including a significantly increased hospital stay and they are 4.5 times less likely to survive to discharge(5). Of those that do survive, patients with deep infection have been found to be three times less likely to return to their original residence (5). These findings have been found in other large cohort studies (6)

Due to the severe consequences of deep infections in this patient group, a number of interventions are undertaken to try and reduce the risk of infection such as perioperative antibiotics, vigorous skin preparation and wound dressing. Wound dressing is one of the most important interventions to address the risk of post-operative infection. The role of a post-operative wound dressing is to minimise bacterial ingress into the wound, manage exudate from the wound site and protect the soft tissues whilst they heal.

Traditionally, the surgical incision is covered with an adhesive dressing or gauze to protect the wound from contamination from the outside environment. These 'standard dressings' have been used throughout the NHS for many years. Negative-pressure wound therapy (NPWT) is an alternative form of dressing which may be applied to closed surgical incisions. In this treatment, an 'open-cell', solid foam overlies the incision and is covered with a semipermeable

membrane which is only permeable to gas. A sealed tube is used to connect the foam to a pump, which creates a partial vacuum over the wound. This negative-pressure therapy provides a sealed environment, preventing bacterial ingress and removes blood and serous fluid exuding the wound. The application of negative pressure to the foam leads to the application of positive pressure to the wound bed and has been shown to reduce the incidence of wound haematoma(7). Recent laboratory studies suggest that NPWT shifts the cytokine profile to being less inflammatory, promotes the production of pro-angiogenic growth factors and enzymes responsible for matrix remodelling, leading to improved wound healing (8-12).

However, a recent Cochrane review for surgical wounds concluded "it is still not clear whether NPWT promotes faster healing and reduces complications associated with clean surgery". "Given the cost and widespread use of NPWT, there is an urgent need for suitably powered, high-quality trials to evaluate the effects of the newer NPWT products that are designed for use on clean, closed surgical incisions. Such trials should focus initially on wounds that may be difficult to heal"(13).

The current feasibility trial will not only assess the feasibility of performing a randomised controlled trial on wound dressings in this challenging population, it will aim to estimate the current infection rates for hip fracture patients. Capture of surgical site infection rates falls under the remit of Public Health England's Surgical Site Infection Surveillance Programme (14). Their report from 2015 reports a surgical site infection incidence of 1.3% (95% CI 1.3%-1.5%)(14). However, infection rates reported in the literature for this patient group are often higher, with some reports stating rates as high as 7% (15, 16). There is growing evidence that the surveillance system from Public Health England is providing a significant underestimate of the true incidence (17-19). This is borne out by local audit of hip fracture patients at two large teaching hospitals in the England, that have found infection rates between 5% and 8% respectively (unpublished data). A reliable estimation of infection will be required to inform the sample size for a large RCT.

**Aims and Objectives**

We aim to conduct a feasibility trial of NPWT versus standard dressing following hip fracture surgery. It is intended to obtain a reliable estimate of the rate of surgical site infections in the target population. In addition, feasibility will be considered in terms of other aspects of the trial such as recruiting and randomising large numbers from individual sites within a relatively short timeframe

For peer review only

The primary objective is:
To quantify differences in the rate of 'deep infection' -as defined by the Centre for Disease Control (CDC)- after hip fracture surgery in standard dressing and NPWT. Patients will be assessed at discharge, 30 days and 120 days. We will examine how many deep infections occur, and when the diagnosis is made.

Both 'how many deep infections' and 'when they are diagnosed' will be crucial to inform the design of a full-scale trial. Each time point addresses the requirements of the CDC, and the routine follow up of patients in the current UK care pathway.

The secondary objectives are:
1) To determine the number and nature of further surgical interventions related to the injury, in the first 4 months after the index procedure
2) To investigate which outcomes will be required to assess cost effectiveness, of negative pressure wound therapy versus standard dressing for wounds associated with hip fracture surgery in a definitive trial.
3) To determine recruitment rate and willingness to participate in the trial.
4) To capture the core outcome set for hip fracture studies

**METHODS AND ANALYSIS**

**Study design**
This is a multicentre two arm unblinded randomised feasibility study embedded within a cohort.

**Setting and Participants**
This feasibility study is embedded within the WHiTE cohort(20). It will be run in 5 different centres across England including Major Trauma Centres and Trauma Units. All patients being surgically treated for hip fracture will be considered for enrolment.

**Eligibility**

Patients will be eligible for this study if:

- They are aged 65 years or older
- They have a hip fracture requiring surgical treatment.

Exclusion criteria: Patients having percutaneous screw fixation of an undisplaced intracapsular fracture of the hip. This small sub-group of patients (less than 5% on the National Hip Fracture Database) have very small surgical incisions -

For peer review only

typically 1-2cm. Where incisions are so small, it is unlikely that they will benefit from an advanced dressing like Negative Pressure Wound Therapy.

**Recruitment of participants, screening and eligibility assessment**

**Consent**

Patients with a hip fracture are a clinical priority for urgent operative care. They will undergo surgery on the next available trauma operating list. All patients with a fracture of the hip are in pain and have received opiate analgesia. It is therefore understandable that the majority of patients find the initial period of their treatment in hospital confusing and disorientating. Similarly, patients' next of kin, carers and friends are often anxious at this time and may have difficulty in weighing the large amounts of information that they are given about the injury and plan for treatment. In this emergency situation the focus is on obtaining consent for surgery (where possible) and informing the patient and any next of kin about immediate clinical care. It is often not possible for the patient or relative/carer (consultee) to review trial documentation, weigh the information and communicate an informed decision about whether they would wish to participate. The consent procedure for this trial will reflect that of the surgery, with the clinical team assessing capacity before taking consent for the surgical procedure and this capacity assessment then being used to decide on the proper approach to consenting to the research. An appropriate method, in line with the mental capacity act and approved by a National Research Ethics Committee, will then be used to gain either prospective or retrospective consent from the patient or appropriate consultee by a GCP-trained, appropriately delegated member of the research team.

**Randomisation**

The treating surgeon will confirm eligibility at the end of the operative procedure but before the wound dressing is applied. Eligible patients will be enrolled into the study via a secure, remote. online randomisation system. To ensure concealment of the allocation sequence, patients will only be randomised after they have been registered on the online system. The allocation sequence will be generated by the trial statistician at the Oxford Clinical Trials Unit. Randomisation will be on a 1:1 basis, stratified by trial centre. All modern operating theatres include a computer with web-access, so a secure, 24-hour, web-based randomisation system will be used to generate the treatment allocation intra-operatively.

**Post randomisation withdrawals/exclusions**

Participants may decline to continue to take part in the trial at any time without prejudice. A decision to decline consent or withdraw will not affect the standard of care the patient receives.

**Blinding**

As the wound dressings are clearly visible, the patients cannot be blind to their treatment. In addition, the treating surgeons will also not be blind to the treatment, but will take no part in the post-operative research assessment of the patients. The clinical

For peer review only

outcome data will be collected by independent research assistants. Questionnaire data will be entered onto the trial central database by adata clerk in the trial central office.

**Interventions**

Patients with a hip fracture usually have surgery on the next available trauma operating list. Some patients may be delayed by medical factors such as anticoagulation, however surgery will proceed as soon as is determined safe by the treating team. All patients will receive a general or regional anaesthetic. Routine prophylactic antibiotics will be given to all patients immediately before surgery and all patients will be assessed for venous thromboembolism prophylaxis according to local policy. At the end of the operation, a dressing is applied to the surgical wound. This trial will involve two types of wound dressing; standard dressing and negative pressure wound therapy.

***Standard dressing.***

The standard dressing for a surgical wound comprises a non-adhesive layer applied directly to the wound which is covered by a sealed dressing. The standard dressing does not use 'negative pressure'. The exact details of the dressing materials and duration will be left to the discretion of the treating surgeon as per their routine practice but the details of each dressing applied in the trial will be recorded.

***Negative-pressure wound therapy.***

The NPWT dressing uses an 'open-cell', solid foam layer which is laid onto the wound as an intrinsic part of a sealed dressing. A sealed tube connects the dressing to a built in mini-pump which creates a partial vacuum over the wound. This can only be kept on for 7 days according to the manufacturer which will be reflected in this study.

Any further wound dressing after the initial dressing will be recorded and will follow the allocated treatment unless otherwise clinically indicated.

**Outcome Measures**

Primary outcome:

The primary outcome measure for this study is deep infection; We will use the Center for Disease Control and Prevention definition of a "deep surgical site infection", that is a wound infection involving the tissues deep to the skin that occurs within 30 or 90 days of injury (21).

The treating clinical team will make the diagnosis of 'deep infection', as per routine clinical practice. The treating clinicians will not be part of the research team. Since the prompt diagnosis and treatment of infection is fundamental to

the patient's routine clinical care, the treating surgeon/clinician will always document such a change in management in the patient's medical record.

In addition to the rate of infection, time of diagnosis will be assessed; The CDC definition applies up to the 90 days after hip fracture surgery(21). However the national care pathway for hip fractures involves remote follow up at 120 days. Therefore to ensure that assessment meets the CDC criteria and is consistent with existing care practice, each patient will be assessed at 30 days and 120 days.

At 30 days the patient will be assessed either as an inpatient or over the telephone according to the relevant clinical parameters.

At 120 days patients will be asked, via telephone interview, about any further symptoms of infection which may have emerged since the 30 day time point. Of the infections that are identified we will establish when the diagnosis was made in the course of recovery.

The secondary outcome measures in this trial are:
***Mortality*** Qualitative work with patients who sustain hip fractures identified mortality as an important metric (22).
***EuroQol EQ-5D-5L***; The EuroQol EQ-5D is a validated measure of health-related quality of life, consisting of a five dimension health status classification system and a separate visual analogue scale. (23) Responses to the health status classification system will be converted into multi-attribute utility (MAU) scores using tariffs currently under development for England (24). These MAU scores will be combined with survival data to generate QALY profiles for the purposes of the economic evaluation. The EQ-5D has been validated to be completed by a patient's proxy in case of continues impaired capacity.
***Complications***; all complications and surgical interventions related to the index wound will be recorded.
***Resource use*** will be monitored to help inform the economic analysis plan in the subsequent definitive trial. Cost data will be obtained from national databases or will be estimated in consultation with the hospital finance department. The cost consequences following discharge, including NHS costs and patients' out-of-pocket expenses will be recorded via a short questionnaire, which will be administered at 4 months post injury. This will be either by patient or consultee(25).
***Mobility*** The ability to walk indoors and outdoors is rated very highly by patients. It has been included in a recommended 'core outcome set' for trials assessing interventions in hip fractures.
***Residential status*** Also captured on CRF. The residential status is also part of the core outcome set for hip fractures and NHFD dataset (22). It will be captured at baseline and at 4 months.

For peer review only - http://bmjopen.bmj.com/site/about/guidelines.xhtml

***Recruitment rate*** The rate of recruitment of potential participants, both in terms of eligibility and those who consent (or on whose behalf consent is provided) will be recorded.

***Retention rate*** The rate of retention through the study visits of participants will be recorded.

**Adverse event management**

Adverse events (AE) are defined as any untoward medical occurrence in a clinical trial subject and which do not necessarily have a causal relationship with the treatment. All AEs will be listed on the appropriate Case Report Form for routine return to the 'WHISH' central office.

Some adverse events are expected as part of the surgical interventions, and do not need to be reported immediately, provided they are recorded in the 'Complications' section of the Case Report Forms and/or Patient Questionnaires. These events are: complications of anaesthesia or surgery (bleeding or damage to adjacent structures such as nerves, tendons and blood vessels, delayed unions/non-unions, further surgery to remove/replace metalwork, dislocation, wound dehiscence and thromboembolic events). All participants experiencing SAEs will be followed-up as per protocol until the end of the trial.

**Follow-up**

Each patient will be assessed in the current UK national audit framework for hip fracture patients. This means they will contact by telephone 4 months (120 days) after surgery. As per the feasibility aims of this study, we will also assess each patient at 30 days. This may be in person as an inpatient or over the telephone is the patient has been discharged.

The 4 month time-point will collect the data required for the National Hip Fracture Database, health-related quality of life and the other components of the hip fracture core outcome set(28). We will also investigate deep infection at the 4 month time point to ensure that each patient has been assessed at 90 days which is required by the CDC.

Details of any late complications will be sent securely to the trial co-ordinating centre. Complications will be captured on the CRF by the site research associate and recorded on the CRFs at 30 days/discharge and at 4 months via telephone interview.

Where a personal consultee has agreed to the patient's involvement we will ask them for the follow up data if the participant has permanently lost capacity to consent. Those patients or consultees who do not wish to provide follow up data

For peer review only - http://bmjopen.bmj.com/site/about/guidelines.xhtml

but agree to the study team reviewing their medical records, clinical reporting forms will be completed by the site and returned in the same manner. This is referred to as 'routine data only' option.

**Sample size**

The purpose of the proposed feasibility study is to determine the rate of deep surgical site infection which in turn will inform the sample size for the definitive trial. We will recruit a sample of 464 participants; divided equally in each of the two study arms. This is based on the number of hip fractures treated at each of the recruiting centres and assuming a conservative recruitment of 50% of eligible patients. 464 is based upon Wilson's confidence interval (CI) method at 2-sided 95% significance level with 20% loss to follow-up). This will allow us to estimate the overall 30 day deep infection rate within 3-5% depending upon the actual rate.

**Rehabilitation**

Early full weight-bearing mobilisation is routine in all cases of hip fracture. Further details of the rehabilitation will be left to the discretion of the treating team as per usual clinical practice.

**Data management**

The Case Report Forms will be designed by the trial manager in conjunction with the trial management team. All electronic patient-identifiable information will be held on a secure, password-protected database at the Kadoorie Centre, accessible only to the research team. Paper forms with patient-identifiable information will be held in secure, locked filing cabinets within a restricted area. Patients will be identified by a code number only. Direct access to source data/documents will be required for trial-related monitoring and/or audit by the Sponsor, NHS Trust or regulatory authorities as required. All paper and electronic data will be retained for at least five years after completion of the trial

**Statistical Analysis**

Standard statistical summaries (e.g. means, standard deviations, medians, IQR) will be reported for all discrete and continuous outcome measures. Baseline data (e.g. age and gender) will be summarised to check comparability between treatment arms. This will only be shared with DMC in the first instance at the end of the feasibility study. No formal hypothesis testing will take place; as per the aim of this feasibility study, the analysis will report the deep infection rates overall and in the two treatment groups on an intention-to-treat basis at discharge or 30 days post-recruitment.

Should this study demonstrate that a full -scale trial is feasible, the data from this feasibility study will be included in the final analysis of the definitive trial. This is contingent on the future definitive trial being sufficiently similar to this

For peer review only

feasibility study. Baseline and follow-up outcome data will therefore not be released to the trial team unless the lack of feasibility is determined based upon observed infection rate at the end of the feasibility study, or the definitive study cannot be conducted for other reasons (e.g. lack of funding). Reporting of the study outcomes will be done accordingly to enable to use of the outcome data in a future definitive trial if such a study goes ahead.

Recruitment and retention rates will be reported through a consort diagram and so will inform the planning of a definitive trial with respects to sample size and the time points of measurements.

It seems likely that some data may not be available due to voluntary withdrawal of patients, lack of completion of individual data items or general loss to follow-up. In this hip fracture cohort there is significant mortality at 30 days-9.3% (1) Where possible the reasons for missing data will be ascertained and reported. Although missing data is not expected to be a problem for this study, the nature and pattern of the missing data is important to understand for the full study, so will be carefully considered.
Reasons for ineligibility, non-compliance, withdrawal or other protocol violations will be stated and any patterns summarized.

**Economic evaluation**

An economic evaluation will not be the primary focus of the trial as it is a feasibility study. However, we will seek to collect relevant health economic data to explore how well this data can be reported in this group and whether any adaptations need to be undertaken to facilitate this. Given the sizable financial difference between the two proposed interventions, a clear understanding of the health economics will be imperative in any subsequent larger trial.

**Trial Oversight**

The day-to-day management of the trial will be the responsibility of the Clinical Trial Manager, based at Nuffield Department of Orthopaedics, Rheumatology and Musculoskeletal Sciences and supported by the OCTRU staff. This will be overseen by the Trial Management Group, who will meet monthly to assess progress. It will also be the responsibility of the Clinical Trial Manager to undertake training of the research associates at each of the trial centres. The Trial Statistician and Health Economist will be closely involved in setting up data capture systems, design of databases and clinical reporting forms.
A Trial Steering Committee (TSC) and a Data & Safety Monitoring Committee (DSMC) will be set up. The study DSMC will adopt a DAMOCLES charter which defines its terms of reference and operation in relation to oversight of the trial. They will not be asked to perform any formal interim analyses of effectiveness. They will, however, see copies of data accrued to date, or summaries of that data by treatment group and they will assess the screening algorithm against the eligibility criteria. They will also consider emerging evidence from other related trials or research and review related SAEs that have been reported. They may advise the chair of the Trial Steering Committee at any time if, in their view, the trial should be stopped for ethical reasons, including concerns about participant safety. DSMC meetings will be held at least annually during the recruitment phase of the study.

For peer review only

**Quality control**

The study may be monitored, or audited in accordance with the current approved protocol, relevant regulations and standard operating procedures by the Host organization, Sponsor or appropriate Regulatory Authorities. A Monitoring Plan will be developed according to Oxford Clinical Trials Research Unit standard operating procedures which involves a risk assessment. The monitoring activities are based on the outcome of the risk assessment and may involve central monitoring and site monitoring**.**

**Ethics and Dissemination**

Ethical approval was granted by Oxford C Research Ethics Committee, 28/04/2017, 17/SC/0207. Results of this study will be disseminated through peer reviewed publication and presentation at conferences.

**References**


**1. Physicians RCo. Falls and Fragility Fracture Audit Programme (FFFAP). National Hip Fracture Database (NHFD) Annual Report 2015. 2015.**

**2. Cooper C, Campion G, Melton LJ, 3rd. Hip fractures in the elderly: a world-wide projection. Osteoporos Int. 1992 Nov;2(6):285-9. PubMed PMID: 1421796. Epub 1992/11/01. eng.**

**3. Johnell O, Kanis JA. An estimate of the worldwide prevalence, mortality and disability associated with hip fracture. Osteoporosis International. 2004 2004/11/01;15(11):897-902. English.**

**4. Roche JJW, Wenn RT, Sahota O, Moran CG. Effect of comorbidities and postoperative complications on mortality after hip fracture in elderly people: prospective observational cohort study. BMJ. 2005 2005-12-08 22:58:01;331(7529):1374.**

**5. Pollard TC, Newman JE, Barlow NJ, Price JD, Willett KM. Deep wound infection after proximal femoral fracture: consequences and costs. J Hosp Infect. 2006 Jun;63(2):133-9. PubMed PMID: 16621145. Epub 2006/04/20. eng.**

**6. Theodorides AA, Pollard TC, Fishlock A, Mataliotakis GI, Kelley T, Thakar C, et al. Treatment of post-operative infections following proximal femoral fractures: our institutional experience. Injury. 2011 Dec;42 Suppl 5:S28-34. PubMed PMID: 22196907. Epub 2011/12/27. eng.**

**7. Stannard JP, Volgas DA, Stewart R, McGwin Jr G, Alonso JE. Negative pressure wound therapy after severe open fractures: A prospective randomized study. Journal of Orthopaedic Trauma. 2009;23(8):552-7.**

**8. Shweiki D, Itin A, Soffer D, Keshet E. Vascular endothelial growth factor induced by hypoxia may mediate hypoxia-initiated angiogenesis. Nature. 1992 Oct 29;359(6398):843-5. PubMed PMID: 1279431. Epub 1992/10/29. eng.**

**9. Saxena V, Hwang CW, Huang S, Eichbaum Q, Ingber D, Orgill DP. Vacuum-assisted closure: microdeformations of wounds and cell proliferation. Plast Reconstr Surg. 2004 Oct;114(5):1086-96; discussion 97-8. PubMed PMID: 15457017. Epub 2004/10/01. eng.**


For peer review only

10. Pietramaggiori G, Liu P, Scherer SS, Kaipainen A, Prsa MJ, Mayer H, et al. Tensile forces stimulate vascular remodeling and epidermal cell proliferation in living skin. Ann Surg. 2007 Nov;246(5):896-902. PubMed PMID: 17968184. Epub 2007/10/31. eng.

11. Morykwas MJ, Argenta LC, Shelton-Brown EI, McGuirt W. Vacuum-assisted closure: a new method for wound control and treatment: animal studies and basic foundation. Ann Plast Surg. 1997 Jun;38(6):553-62. PubMed PMID: 9188970. Epub 1997/06/01. eng.

12. Pollak AN. Use of negative pressure wound therapy with reticulated open cell foam for lower extremity trauma. J Orthop Trauma. 2008 Nov-Dec;22(10 Suppl):S142-5. PubMed PMID: 19034161. Epub 2008/12/17. eng.

13. Webster J, Scuffham P, Stankiewicz M, Chaboyer WP. Negative pressure wound therapy for skin grafts and surgical wounds healing by primary intention. Cochrane Database Syst Rev. 2014;10:Cd009261. PubMed PMID: 25287701. Epub 2014/10/08. eng.

14. England PH. Surveillance of surgical site infections in NHS hospitals in England: 2014 to 2015. 2015.

15. Noailles T, Brulefert K, Chalopin A, Longis PM, Gouin F. What are the risk factors for post-operative infection after hip hemiarthroplasty? Systematic review of literature. Int Orthop. 2015 Nov 27. PubMed PMID: 26611729. Epub 2015/11/28. Eng.

16. Edwards C, Counsell A, Boulton C, Moran CG. Early infection after hip fracture surgery: RISK FACTORS, COSTS AND OUTCOME. Journal of Bone & Joint Surgery, British Volume. 2008;90-B(6):770-7.

17. Wilson J, Charlett A, Leong G, McDougall C, Duckworth G. Rates of surgical site infection after hip replacement as a hospital performance indicator: analysis of data from the English mandatory surveillance system. Infect Control Hosp Epidemiol. 2008 Mar;29(3):219-26. PubMed PMID: 18257691. Epub 2008/02/09. eng.

18. Singh S, Davies J, Sabou S, Shrivastava R, Reddy S. Challenges in reporting surgical site infections to the national surgical site infection surveillance and suggestions for improvement. Ann R Coll Surg Engl. 2015 Sep;97(6):460-5. PubMed PMID: 26320763. Epub 2015/09/01. eng.

19. Tanner J, Padley W, Kiernan M, Leaper D, Norrie P, Baggott R. A benchmark too far: findings from a national survey of surgical site infection surveillance. J Hosp Infect. 2013 Feb;83(2):87-91. PubMed PMID: 23332352. Epub 2013/01/22. eng.

20. Costa ML, Griffin XL, Achten J, Metcalfe D, Judge A, Pinedo-Villanueva R, et al. World Hip Trauma Evaluation (WHiTE): framework for embedded comprehensive cohort studies. BMJ Open. 2016;6(10).

21. Horan TC, Andrus M, Dudeck MA. CDC/NHSN surveillance definition of health care-associated infection and criteria for specific types of infections in the acute care setting. Am J Infect Control. 2008 Jun;36(5):309-32. PubMed PMID: 18538699. Epub 2008/06/10. eng.

22. Haywood KL, Griffin XL, Achten J, Costa ML. Developing a core outcome set for hip fracture trials. Bone Joint J. 2014 Aug;96-b(8):1016-23. PubMed PMID: 25086115. Epub 2014/08/03. eng.

23. Brooks R. EuroQol: the current state of play. Health Policy. 1996 Jul;37(1):53-72. PubMed PMID: 10158943. Epub 1996/06/06. eng.

For peer review only

**24. Oppe M, Devlin NJ, van Hout B, Krabbe PF, de Charro F. A program of methodological research to arrive at the new international EQ-5D-5L valuation protocol. Value Health. 2014 Jun;17(4):445-53. PubMed PMID: 24969006.**
**25. Self-reports of health care utilisation: can a questionnaire replace a diary? 16th Annual meeting of the international society for health technology assessment in health care; 2000; The Hague, The Netherlands.**

**Authors Contributions**

JM/MC/JA wrote the background section and developed the research question. JM/MC/JA/LS were responsible for the research methodology and management. JC wrote the sample size and statistical analysis sections of the protocol. MD wrote the health economic evaluation section of the protocol.
All authors reviewed and agreed the final manuscript.

**Competing interest**

No relevant competing interests to declare. ICMJE forms for each author held by corresponding author.

**Funding statement**

The study is funded by the Royal College of Surgeons of England and Dunhill Medical Trust and supported by the National Institute for Health Research (NIHR) Oxford Biomedical Research Centre. Excess treatment costs for the negative pressure wound therapy arm of the study are met by Smith and Nephew.

For peer review only

SPIRIT
STANDARD PROTOCOL ITEMS: RECOMMENDATIONS FOR INTERVENTIONAL TRIALS

SPIRIT 2013 Checklist: Recommended items to address in a clinical trial protocol and related documents*

| Section/item | Item No | Description | Addressed on page number |
|---|---|---|---|
| **Administrative information** | | | |
| Title | 1 | Descriptive title identifying the study design, population, interventions, and, if applicable, trial acronym | ________1______ |
| Trial registration | 2a | Trial identifier and registry name. If not yet registered, name of intended registry | _____2_________ |
| | 2b | All items from the World Health Organization Trial Registration Data Set | _____2_________ |
| Protocol version | 3 | Date and version identifier | _____1________ |
| Funding | 4 | Sources and types of financial, material, and other support | _______15_______ |
| Roles and responsibilities | 5a | Names, affiliations, and roles of protocol contributors | ____1__________ |
| | 5b | Name and contact information for the trial sponsor | ____1________ |
| | 5c | Role of study sponsor and funders, if any, in study design; collection, management, analysis, and interpretation of data; writing of the report; and the decision to submit the report for publication, including whether they will have ultimate authority over any of these activities | _____15_________ |
| | 5d | Composition, roles, and responsibilities of the coordinating centre, steering committee, endpoint adjudication committee, data management team, and other individuals or groups overseeing the trial, if applicable (see Item 21a for data monitoring committee) | _____15______ |



For peer review only - http://bmjopen.bmj.com/site/about/guidelines.xhtml

For peer review only

**Introduction**

| | | | |
|---|---|---|---|
| Background and rationale | 6a | Description of research question and justification for undertaking the trial, including summary of relevant studies (published and unpublished) examining benefits and harms for each intervention | __4________ |
| | 6b | Explanation for choice of comparators | ___5________ |
| Objectives | 7 | Specific objectives or hypotheses | ___5________ |
| Trial design | 8 | Description of trial design including type of trial (eg, parallel group, crossover, factorial, single group), allocation ratio, and framework (eg, superiority, equivalence, noninferiority, exploratory) | ___6_______ |

**Methods: Participants, interventions, and outcomes**

| | | | |
|---|---|---|---|
| Study setting | 9 | Description of study settings (eg, community clinic, academic hospital) and list of countries where data will be collected. Reference to where list of study sites can be obtained | ___6________ |
| Eligibility criteria | 10 | Inclusion and exclusion criteria for participants. If applicable, eligibility criteria for study centres and individuals who will perform the interventions (eg, surgeons, psychotherapists) | _6-7_________ |
| Interventions | 11a | Interventions for each group with sufficient detail to allow replication, including how and when they will be administered | ___8________ |
| | 11b | Criteria for discontinuing or modifying allocated interventions for a given trial participant (eg, drug dose change in response to harms, participant request, or improving/worsening disease) | __12_______ |
| | 11c | Strategies to improve adherence to intervention protocols, and any procedures for monitoring adherence (eg, drug tablet return, laboratory tests) | ___N/A________ |
| | 11d | Relevant concomitant care and interventions that are permitted or prohibited during the trial | ___8________ |
| Outcomes | 12 | Primary, secondary, and other outcomes, including the specific measurement variable (eg, systolic blood pressure), analysis metric (eg, change from baseline, final value, time to event), method of aggregation (eg, median, proportion), and time point for each outcome. Explanation of the clinical relevance of chosen efficacy and harm outcomes is strongly recommended | ___8-10_____ |
| Participant timeline | 13 | Time schedule of enrolment, interventions (including any run-ins and washouts), assessments, and visits for participants. A schematic diagram is highly recommended (see Figure) | __10________ |



For peer review only - http://bmjopen.bmj.com/site/about/guidelines.xhtml

| | | | |
|---|---|---|---|
| Sample size | 14 | Estimated number of participants needed to achieve study objectives and how it was determined, including clinical and statistical assumptions supporting any sample size calculations | _____11____ |
| Recruitment | 15 | Strategies for achieving adequate participant enrolment to reach target sample size | ____11____ |
| **Methods: Assignment of interventions (for controlled trials)** | | | |
| Allocation: | | | |
| Sequence generation | 16a | Method of generating the allocation sequence (eg, computer-generated random numbers), and list of any factors for stratification. To reduce predictability of a random sequence, details of any planned restriction (eg, blocking) should be provided in a separate document that is unavailable to those who enrol participants or assign interventions | ___7________ |
| Allocation concealment mechanism | 16b | Mechanism of implementing the allocation sequence (eg, central telephone; sequentially numbered, opaque, sealed envelopes), describing any steps to conceal the sequence until interventions are assigned | __7________ |
| Implementation | 16c | Who will generate the allocation sequence, who will enrol participants, and who will assign participants to interventions | __7________ |
| Blinding (masking) | 17a | Who will be blinded after assignment to interventions (eg, trial participants, care providers, outcome assessors, data analysts), and how | __7_________ |
| | 17b | If blinded, circumstances under which unblinding is permissible, and procedure for revealing a participant's allocated intervention during the trial | ____7_______ |
| **Methods: Data collection, management, and analysis** | | | |
| Data collection methods | 18a | Plans for assessment and collection of outcome, baseline, and other trial data, including any related processes to promote data quality (eg, duplicate measurements, training of assessors) and a description of study instruments (eg, questionnaires, laboratory tests) along with their reliability and validity, if known. Reference to where data collection forms can be found, if not in the protocol | _10-11_______ |
| | 18b | Plans to promote participant retention and complete follow-up, including list of any outcome data to be collected for participants who discontinue or deviate from intervention protocols | __10-11_______ |



For peer review only - http://bmjopen.bmj.com/site/about/guidelines.xhtml

| | | | |
|---|---|---|---|
| Data management | 19 | Plans for data entry, coding, security, and storage, including any related processes to promote data quality (eg, double data entry; range checks for data values). Reference to where details of data management procedures can be found, if not in the protocol | ___11_______ |
| Statistical methods | 20a | Statistical methods for analysing primary and secondary outcomes. Reference to where other details of the statistical analysis plan can be found, if not in the protocol | __11-12____ |
| | 20b | Methods for any additional analyses (eg, subgroup and adjusted analyses) | ___11-12____ |
| | 20c | Definition of analysis population relating to protocol non-adherence (eg, as randomised analysis), and any statistical methods to handle missing data (eg, multiple imputation) | __11-12_____ |
| **Methods: Monitoring** | | | |
| Data monitoring | 21a | Composition of data monitoring committee (DMC); summary of its role and reporting structure; statement of whether it is independent from the sponsor and competing interests; and reference to where further details about its charter can be found, if not in the protocol. Alternatively, an explanation of why a DMC is not needed | ___12_____ |
| | 21b | Description of any interim analyses and stopping guidelines, including who will have access to these interim results and make the final decision to terminate the trial | ____12______ |
| Harms | 22 | Plans for collecting, assessing, reporting, and managing solicited and spontaneously reported adverse events and other unintended effects of trial interventions or trial conduct | _____10_____ |
| Auditing | 23 | Frequency and procedures for auditing trial conduct, if any, and whether the process will be independent from investigators and the sponsor | _13____ |
| **Ethics and dissemination** | | | |
| Research ethics approval | 24 | Plans for seeking research ethics committee/institutional review board (REC/IRB) approval | ___13_____ |
| Protocol amendments | 25 | Plans for communicating important protocol modifications (eg, changes to eligibility criteria, outcomes, analyses) to relevant parties (eg, investigators, REC/IRBs, trial participants, trial registries, journals, regulators) | __13_____ |



For peer review only - http://bmjopen.bmj.com/site/about/guidelines.xhtml

| | | | |
|---|---|---|---|
| Consent or assent | 26a | Who will obtain informed consent or assent from potential trial participants or authorised surrogates, and how (see Item 32) | ____7______ |
| | 26b | Additional consent provisions for collection and use of participant data and biological specimens in ancillary studies, if applicable | ____N/A______ |
| Confidentiality | 27 | How personal information about potential and enrolled participants will be collected, shared, and maintained in order to protect confidentiality before, during, and after the trial | ____11______ |
| Declaration of interests | 28 | Financial and other competing interests for principal investigators for the overall trial and each study site | Application |
| Access to data | 29 | Statement of who will have access to the final trial dataset, and disclosure of contractual agreements that limit such access for investigators | ___13______ |
| Ancillary and post-trial care | 30 | Provisions, if any, for ancillary and post-trial care, and for compensation to those who suffer harm from trial participation | __10______ |
| Dissemination policy | 31a | Plans for investigators and sponsor to communicate trial results to participants, healthcare professionals, the public, and other relevant groups (eg, via publication, reporting in results databases, or other data sharing arrangements), including any publication restrictions | __13_______ |
| | 31b | Authorship eligibility guidelines and any intended use of professional writers | _____N/A______ |
| | 31c | Plans, if any, for granting public access to the full protocol, participant-level dataset, and statistical code | ___13_______ |
| **Appendices** | | | |
| Informed consent materials | 32 | Model consent form and other related documentation given to participants and authorised surrogates | ____Appendix |
| Biological specimens | 33 | Plans for collection, laboratory evaluation, and storage of biological specimens for genetic or molecular analysis in the current trial and for future use in ancillary studies, if applicable | __N/A________ |

*It is strongly recommended that this checklist be read in conjunction with the SPIRIT 2013 Explanation & Elaboration for important clarification on the items. Amendments to the protocol should be tracked and dated. The SPIRIT checklist is copyrighted by the SPIRIT Group under the Creative Commons "Attribution-NonCommercial-NoDerivs 3.0 Unported" license.



For peer review only - http://bmjopen.bmj.com/site/about/guidelines.xhtml

BMJ Open

# WHITE 7 - WHISH – Wound Healing in Surgery for Hip fractures
# A Randomised Controlled Feasibility Trial of standard wound management versus negative pressure wound therapy in the treatment of adult patients having surgical incisions for hip fractures


| | |
|---|---|
| Journal: | *BMJ Open* |
| Manuscript ID | bmjopen-2017-020632.R1 |
| Article Type: | Protocol |
| Date Submitted by the Author: | 24-Jan-2018 |
| Complete List of Authors: | Masters, James; University of Oxford, Oxford Trauma, Nuffield Department of Orthopaedics, Rheumatology and Musculoskeletal sciences (NDORMS)<br>Achten, Juul; University of Oxford, NDORMS<br>Cook, Jonathan; University of Oxford, NDORMS<br>Dritsaki, Melina; University of Oxford Nuffield Department of Orthopaedics Rheumatology and Musculoskeletal Sciences,<br>Sansom, Lucy; University of Oxford, Nuffield Department of Orthopaedics, Rheumatology, and Musculoskeletal Sciences<br>Costa, Matthew; University of Oxford, Nuffield Department of Orthopaedics, Rheumatology, and Musculoskeletal Sciences |
| <b>Primary Subject Heading</b>: | Surgery |
| Secondary Subject Heading: | Infectious diseases |
| Keywords: | Hip < ORTHOPAEDIC & TRAUMA SURGERY, surgical site infection, hospital acquired infection |
| | |


SCHOLARONE™
Manuscripts

# WHITE 7 - WHISH – Wound Healing in Surgery for Hip fractures A Randomised Controlled Feasibility Trial of standard wound management versus negative pressure wound therapy in the treatment of adult patients having surgical incisions for hip fractures.

Corresponding author
James Masters
Oxford Trauma, NDORMS
University of Oxford
Oxford, OX3 9DU
James.masters@ndorms.ox.ac.uk
01865 223114

James Masters[1] Juul Achten[1], Jonathan Cook[2], Melina Dritsaki[3], Lucy Sansom[1] and Matthew L Costa[1].

[1] Oxford Trauma, Nuffield Department of Orthopaedics, Rheumatology & Musculoskeletal Sciences, University of Oxford, Oxford, UK
[2] Centre for Statistics in Medicine, Nuffield Department of Orthopaedics, Rheumatology & Musculoskeletal Sciences, University of Oxford, Oxford, UK
[3] Oxford Clinical Trial Unit, Nuffield Department of Orthopaedics, Rheumatology & Musculoskeletal Sciences, University of Oxford, Oxford, UK

Word Count: 3856 Based on version number 4.0 1st November 2017

For peer review only

**Abstract**

**Introduction**

Deep wound infection is a catastrophic complication after hip fracture surgery. However, current understanding of infection rates in this population is limited. Many technologies such as incisional negative pressure wound therapy (NPWT) show promise in reducing the rate of infection. This trial is a feasibility study looking to establish a value estimated with a greater precision of the rate of deep infection after hip fracture treatment in patients treated with NPWT versus standard dressing following hip fracture surgery.

**Methods and Analysis**

A randomised controlled trial of 464 patients will be run across multiple centres. It is embedded in the World Hip Trauma Evaluation cohort study. Any patient over the age of 65 years having surgery for hip fracture is eligible unless they are being treated with percutaneous screw fixation. A web based randomisation sequence will stratify patients by centre. Patients will be allocated to either NPWT or standard care on a 1:1 basis. The primary outcome measure is the Centre for Disease Control definition of deep infection at 30 days. Follow up at 4 months will also assess deep infection and the core outcome dataset for hip fractures. This includes health related quality of life (EQ-5D-5L), mobility, mortality and late complications such as further surgery. The primary analysis will be intention to treat.

**Ethics and Dissemination**

Oxford C Research Ethics Committee granted ethical approval on 28/04/2017, 17/SC/0207. This results of this study will be reported in a peer reviewed publication and inform the design of a future full-scale trial.

**Trial registration number**

ISRCTN55305726;

Abstract word Count: 252

For peer review only

Strengths and limitations of the study

- This prospective trial will estimate the true rate of infection according to nationally recognised criteria.
- Robust data collection systems will be used to determine the rate of deep infection.
- This study will include the full range of hip fracture patients including those without capacity, across multiple centres ensuring good external validity to our findings.
- The potential limitation will be to accurately capture those cases of infection where patients move through different institutions and care pathways.

For peer review only

**Introduction**

Hip fracture is one of the biggest challenges facing patients and healthcare systems at present. Worldwide there are 1.3 million hip fractures with more than 65,000 hip fractures in England, Wales and Northern Ireland every year (1). These figures are projected to rise to >100,000 by 2020 in the UK (2) and more than 6 million by 2050 worldwide (3). The global cost of this clinical problem is estimated at 1.75 million disability adjusted life years lost and represents 1.4% of the total healthcare burden in established market economies (4).

The overwhelming majority of hip fractures are treated surgically. The principal objective of surgical treatment is to enable patients to return to weight bearing mobilisation as soon as possible.

As with all surgical treatments, one of the most important potential complications is surgical site infection (SSI). Hip fracture patients are particularly vulnerable to wound problems/surgical site infection. The reasons for this vulnerability are multifactorial. For instance, sixty percent of hip fracture patients have at least one major medical co-morbidity such as steroid or other immunosuppressive medication use, malignancy and diabetes (5). These co-morbidities sit alongside age related deterioration of immune function.

Surgical site infection can be either superficial or deep. Superficial infections only involve the skin and subcutaneous tissue, whereas 'deep' infections involve the deeper layers and the metalwork implanted in the hip. Deep infection has profound consequences for hip fracture patients including a significantly increased hospital stay and they are 4.5 times less likely to survive to discharge (6). Of those that do survive, patients with deep infection have been found to be three times less likely to return to their original residence (6). These findings have been found in other large cohort studies (7).

Due to the severe consequences of deep infections in this patient group, a number of interventions are undertaken to try and reduce the risk of infection such as perioperative antibiotics, vigorous skin preparation and wound dressing. Wound dressing is one of the most important interventions to address the risk of post-operative infection. The role of a post-operative wound dressing is to minimise bacterial ingress into the wound, manage exudate from the wound site and protect the soft tissues whilst they heal.

Traditionally, the surgical incision is covered with an adhesive dressing or gauze to protect the wound from contamination from the outside environment. These 'standard dressings' have been used throughout the NHS for many years. Negative-pressure wound therapy (NPWT) is an alternative form of dressing

For peer review only

which may be applied to closed surgical incisions. In this treatment, an 'open-cell', solid foam overlies the incision and is covered with a semipermeable membrane which is only permeable to gas. A sealed tube is used to connect the foam to a pump, which creates a partial vacuum over the wound. This negative-pressure therapy provides a sealed environment, preventing bacterial ingress and removes blood and serous fluid exuding the wound. The application of negative pressure to the foam leads to the application of positive pressure to the wound bed and has been shown to reduce the incidence of wound haematoma (8). Recent laboratory studies suggest that NPWT shifts the cytokine profile to being less inflammatory, promotes the production of pro-angiogenic growth factors and enzymes responsible for matrix remodelling, leading to improved wound healing (9-13).

However, a recent Cochrane review for surgical wounds concluded "it is still not clear whether NPWT promotes faster healing and reduces complications associated with clean surgery". "Given the cost and widespread use of NPWT, there is an urgent need for suitably powered, high-quality trials to evaluate the effects of the newer NPWT products that are designed for use on clean, closed surgical incisions. Such trials should focus initially on wounds that may be difficult to heal" (14).

The current feasibility trial will not only assess the feasibility of performing a randomised controlled trial on wound dressings in this challenging population, it will aim to estimate the current infection rates for hip fracture patients. Capture of surgical site infection rates falls under the remit of Public Health England's Surgical Site Infection Surveillance Programme (15). Their report from 2015 reports a surgical site infection incidence of 1.3% (95% CI 1.3%-1.5%)(15). However, infection rates reported in the literature for this patient group are often higher, with some reports stating rates as high as 7% (16, 17). There is growing evidence that the surveillance system from Public Health England is providing a significant underestimate of the true incidence (18-20). This is borne out by local audit of hip fracture patients at two large teaching hospitals in the England, that have found infection rates between 5% and 8% respectively (unpublished data). A reliable estimation of infection will be required to inform the sample size for a large RCT.

**Aims and Objectives**

We aim to conduct a feasibility trial of NPWT versus standard dressing following hip fracture surgery. It is intended to obtain a reliable estimate of the rate of surgical site infections in the target population. In addition, feasibility will be considered in terms of other aspects of the trial such as recruiting and randomising large numbers from individual sites within a relatively short timeframe.

For peer review only - http://bmjopen.bmj.com/site/about/guidelines.xhtml

The primary objective is:
To quantify differences in the rate of 'deep infection' -as defined by the Centre for Disease Control (CDC)- after hip fracture surgery in standard dressing and NPWT. Patients will be assessed at 30 days and 4 months. We will examine how many deep infections occur, and when the diagnosis is made.

Both 'how many deep infections' and 'when they are diagnosed' will be crucial to inform the design of a full-scale trial. Each time point addresses the requirements of the CDC, and the routine follow up of patients in the current UK care pathway.

The secondary objectives are:
1) To determine the number and nature of further surgical interventions related to the injury, in the first 4 months after the index procedure.
2) To investigate which outcomes will be required to assess cost effectiveness, of negative pressure wound therapy versus standard dressing for wounds associated with hip fracture surgery in a definitive trial.
3) To determine recruitment rate and willingness to participate in the trial.
4) To capture the core outcome set for hip fracture studies.

## METHODS AND ANALYSIS

### Study design

This is a multicentre two arm unblinded randomised feasibility study embedded within a prospective cohort.

### Setting and Participants

This feasibility study is embedded within the WHiTE cohort (21). It will be run in 5 different centres across England including Major Trauma Centres and Trauma Units. All patients being surgically treated for hip fracture will be considered for enrolment.

### Eligibility

Patients will be eligible for this study if:

- They are aged 65 years or older
- They have a hip fracture requiring surgical treatment.

For peer review only - http://bmjopen.bmj.com/site/about/guidelines.xhtml

Exclusion criteria: Patients having percutaneous screw fixation of an undisplaced intracapsular fracture of the hip. This small sub-group of patients (less than 5% on the National Hip Fracture Database) have very small surgical incisions - typically 1-2cm. Where incisions are so small, it is unlikely that they will benefit from an advanced dressing like Negative Pressure Wound Therapy.

**Recruitment of participants, screening and eligibility assessment**

**Consent**

Patients with a hip fracture are a clinical priority for urgent operative care. They will undergo surgery on the next available trauma operating list. All patients with a fracture of the hip are in pain and have received opiate analgesia. It is therefore understandable that the majority of patients find the initial period of their treatment in hospital confusing and disorientating. Similarly, patients' next of kin, carers and friends are often anxious at this time and may have difficulty in weighing the large amounts of information that they are given about the injury and plan for treatment. In this emergency situation the focus is on obtaining consent for surgery (where possible) and informing the patient and any next of kin about immediate clinical care. This is supported by national guidance that recommends surgery should take place within 36 hours (22) . It is often not possible for the patient or relative/carer (consultee) to review trial documentation, weigh the information and communicate an informed decision about whether they would wish to participate. The consent procedure for this trial will reflect that of the surgery, with the clinical team assessing capacity before taking consent for the surgical procedure and this capacity assessment then being used to decide on the proper approach to consenting to the research. An appropriate method, in line with the mental capacity act and approved by a National Research Ethics Committee, will then be used to gain either prospective or retrospective consent from the patient or appropriate consultee by a GCP-trained, appropriately delegated member of the research team.

**Randomisation**

The treating surgeon will confirm eligibility at the end of the operative procedure but before the wound dressing is applied. Eligible patients will be enrolled into the study via a secure, remote online randomisation system. To ensure concealment of the allocation sequence, patients will only be randomised after they have been registered on the online system. The allocation sequence will be generated by the trial statistician at the Oxford Clinical Trials Unit. Randomisation will be on a 1:1 basis, stratified by trial centre. All modern operating theatres include a computer with web-access, so a secure, 24-hour, web-based randomisation system will be used to generate the treatment allocation intra-operatively.

For peer review only

**Post randomisation withdrawals/exclusions**

Participants may decline to continue to take part in the trial at any time without prejudice. A decision to decline consent or withdraw will not affect the standard of care the patient receives.

**Blinding**

As the wound dressings are clearly visible, the patients cannot be blind to their treatment. In addition, the treating surgeons will also not be blind to the treatment, but will take no part in the post-operative research assessment of the patients. The clinical outcome data will be collected by independent research assistants who will not be blind to the treatment allocation. Questionnaire data will be entered onto the trial central database by a data clerk in the trial central office.

**Interventions**

Patients with a hip fracture usually have surgery on the next available trauma operating list. Some patients may be delayed by medical factors such as anticoagulation, however surgery will proceed as soon as is determined safe by the treating team. All patients will receive a general or regional anaesthetic. Routine prophylactic antibiotics will be given to all patients immediately before surgery and all patients will be assessed for venous thromboembolism prophylaxis according to local policy. At the end of the operation, a dressing is applied to the surgical wound. This trial will involve two types of wound dressing; standard dressing and negative pressure wound therapy.

***Standard dressing.***

The standard dressing for a surgical wound comprises a non-adhesive layer applied directly to the wound that is covered by a sealed dressing. The standard dressing does not use 'negative pressure'. The exact details of the dressing materials and duration will be left to the discretion of the treating surgeon as per their routine practice but the details of each dressing applied in the trial will be recorded.

***Negative-pressure wound therapy***

The NPWT dressing uses an 'open-cell', solid foam layer which is laid onto the wound as an intrinsic part of a sealed dressing. A sealed tube connects the dressing to a built in mini-pump that creates a partial vacuum over the wound. This can only be kept on for 7 days according to the manufacturer that will be reflected in this study.

For peer review only - http://bmjopen.bmj.com/site/about/guidelines.xhtml

Any further wound dressing after the initial dressing will be recorded and will follow the allocated treatment unless otherwise clinically indicated.

**Outcome Measures**

Primary outcome:

The primary outcome measure for this study is deep infection; We will use the Center for Disease Control and Prevention definition of a “deep surgical site infection”, that is a wound infection involving the tissues deep to the skin that occurs within 30 or 90 days of injury (23).

The treating clinical team will make the diagnosis of ‘deep infection’, as per routine clinical practice. The treating clinicians will not be part of the research team. Since the prompt diagnosis and treatment of infection is fundamental to the patient’s routine clinical care, the treating surgeon/clinician will always document such a change in management in the patient’s medical record.

In addition to the rate of infection, time of diagnosis will be assessed; The CDC definition applies up to the 90 days after hip fracture surgery (23). However the national care pathway for hip fractures involves remote follow up at 4 months. Therefore to ensure that assessment meets the CDC criteria and is consistent with existing care practice, each patient will be assessed at 30 days and 4 months.

At 30 days the patient will be assessed either as an inpatient or over the telephone according to the relevant clinical parameters.

At 4 months patients will be asked, via telephone interview, about any further symptoms of infection which may have emerged since the 30 day time point. Of the infections that are identified we will establish when the diagnosis was made in the course of recovery.

The secondary outcome measures in this trial are:

***Mortality*** Qualitative work with patients who sustain hip fractures identified mortality as an important metric (24).

***EuroQol EQ-5D-5L*** The EuroQol EQ-5D is a validated measure of health-related quality of life, consisting of a five dimension health status classification system and a separate visual analogue scale (25). Responses to the health status classification system will be converted into multi-attribute utility (MAU) scores using tariffs currently under development for England (26). These MAU scores will be combined with survival data to generate QALY profiles for the purposes

For peer review only

398 of the economic evaluation. The EQ-5D has been validated to be completed by a
399 patient's proxy in case of continues impaired capacity.
400 ***Complications*** all complications and surgical interventions related to the index
401 wound will be recorded.
402 ***Resource use*** will be monitored to help inform the economic analysis plan in the
403 subsequent definitive trial. Cost data will be obtained from national databases
404 or will be estimated in consultation with the hospital finance department. The
405 cost consequences following discharge, including NHS costs and patients' out-of-
406 pocket expenses will be recorded via a short questionnaire, which will be
407 administered at 4 months post injury. This will be either by patient or consultee
408 (27).
409 ***Mobility*** The ability to walk indoors and outdoors is rated very highly by
410 patients. It has been included in a recommended 'core outcome set' for trials
411 assessing interventions in hip fractures.
412 ***Residential status*** Also captured on CRF. The residential status is also part of
413 the core outcome set for hip fractures and NHFD dataset (24). It will be captured
414 at baseline and at 4 months.
415 ***Recruitment rate*** The rate of recruitment of potential participants, both in
416 terms of eligibility and those who consent (or on whose behalf consent is
417 provided) will be recorded.
418 ***Retention rate*** The rate of retention through the study visits of participants will
419 be recorded.
420
421 **Adverse event management**
422 Adverse events (AE) are defined as any untoward medical occurrence in a
423 clinical trial subject and which do not necessarily have a causal relationship with
424 the treatment. All AEs will be listed on the appropriate Case Report Form for
425 routine return to the 'WHISH' central office.
426 Some adverse events are expected as part of the surgical interventions, and do
427 not need to be reported immediately, provided they are recorded in the
428 'Complications' section of the Case Report Forms and/or Patient Questionnaires.
429 These events are: complications of anaesthesia or surgery (bleeding or damage
430 to adjacent structures such as nerves, tendons and blood vessels, delayed
431 unions/non-unions, further surgery to remove/replace metalwork, dislocation,
432 wound dehiscence and thromboembolic events). All participants experiencing
433 SAEs will be followed-up as per protocol until the end of the trial.
434
435 **Follow-up**
436 Each patient will be assessed in the current UK national audit framework for hip
437 fracture patients. This means they will contact by telephone 4 months after
438 surgery. As per the feasibility aims of this study, we will also assess each patient
439 at 30 days. This may be in person as an inpatient or over the telephone is the
440 patient has been discharged.

For peer review only

The 4 month time-point will collect the data required for the National Hip Fracture Database, health-related quality of life and the other components of the hip fracture core outcome set (24). We will also investigate deep infection at the 4 month time point to ensure that each patient has been assessed by 90 days which is required by the CDC.

Details of any late complications will be sent securely to the trial co-ordinating centre. Complications will be captured on the CRF by the site research associate and recorded on the CRFs at 30 days/discharge and at 4 months via telephone interview.

Where a personal consultee has agreed to the patient's involvement we will ask them for the follow up data if the participant has permanently lost capacity to consent. Those patients or consultees who do not wish to provide follow up data but agree to the study team reviewing their medical records, clinical reporting forms will be completed by the site and returned in the same manner. This is referred to as 'routine data only' option.

**Sample size**

The purpose of the proposed feasibility study is to determine the rate of deep surgical site infection which in turn will inform the sample size for the definitive trial. We will recruit a sample of 464 participants; divided equally in each of the two study arms. This is based on the number of hip fractures treated at each of the recruiting centres and assuming a conservative recruitment of 50% of eligible patients. 464 is based upon Wilson's confidence interval (CI) method at 2-sided 95% significance level with 20% loss to follow-up). This will allow us to estimate the overall 30 day deep infection rate within 3-5% depending upon the actual rate.

**Rehabilitation**

Early full weight-bearing mobilisation is routine in all cases of hip fracture. Further details of the rehabilitation will be left to the discretion of the treating team as per usual clinical practice.

**Data management**

The Case Report Forms will be designed by the trial manager in conjunction with the trial management team. All electronic patient-identifiable information will be held on a secure, password-protected database at the Kadoorie Centre, accessible only to the research team. Paper forms with patient-identifiable information will be held in secure, locked filing cabinets within a restricted area. Patients will be identified by a code number only. Direct access to source data/documents will be required for trial-related monitoring and/or audit by the

For peer review only

Sponsor, NHS Trust or regulatory authorities as required. All paper and electronic data will be retained for at least five years after completion of the trial

**Statistical Analysis**

Standard statistical summaries (e.g. means, standard deviations, medians, IQR) will be reported for all discrete and continuous outcome measures. Baseline data (e.g. age and gender) will be summarised to check comparability between treatment arms. This will only be shared with DMC in the first instance at the end of the feasibility study. No formal hypothesis testing will take place; as per the aim of this feasibility study, the analysis will report the deep infection rates overall and in the two treatment groups on an intention-to-treat basis at 30 days and 4 months post-recruitment.

Should this study demonstrate that a full -scale trial is feasible, the data from this feasibility study will be included in the final analysis of the definitive trial. This is contingent on the future definitive trial being sufficiently similar to this feasibility study. Baseline and follow-up outcome data will therefore not be released to the trial team unless the lack of feasibility is determined based upon observed infection rate at the end of the feasibility study, or the definitive study cannot be conducted for other reasons (e.g. lack of funding). Reporting of the study outcomes will be done accordingly to enable to use of the outcome data in a future definitive trial if such a study goes ahead.

Recruitment and retention rates will be reported through a consort diagram and so will inform the planning of a definitive trial with respects to sample size and the time points of measurements.

It seems likely that some data may not be available due to voluntary withdrawal of patients, lack of completion of individual data items or general loss to follow-up. In this hip fracture cohort there is significant mortality at 30 days-6.7% (1). Where possible the reasons for missing data will be ascertained and reported. Although missing data is not expected to be a problem for this study, the nature and pattern of the missing data is important to understand for the full study, so will be carefully considered.
Reasons for ineligibility, non-compliance, withdrawal or other protocol violations will be stated and any patterns summarized.

**Economic evaluation**

An economic evaluation will not be the primary focus of the trial as it is a feasibility study. However, we will seek to collect relevant health economic data to explore how well this data can be reported in this group and whether any adaptations need to be undertaken to facilitate this. Given the sizable financial difference between the two proposed interventions, a clear understanding of the health economics will be imperative in any subsequent larger trial.

For peer review only

**Trial Oversight**

The day-to-day management of the trial will be the responsibility of the Clinical Trial Manager, based at Nuffield Department of Orthopaedics, Rheumatology and Musculoskeletal Sciences and supported by the OCTRU staff. This will be overseen by the Trial Management Group, who will meet monthly to assess progress. It will also be the responsibility of the Clinical Trial Manager to undertake training of the research associates at each of the trial centres. The Trial Statistician and Health Economist will be closely involved in setting up data capture systems, design of databases and clinical reporting forms.

A Trial Steering Committee (TSC) and a Data & Safety Monitoring Committee (DSMC) will be set up. The study DSMC will adopt a DAMOCLES charter which outlines its responsibilities. They will not perform any formal interim analyses of effectiveness. They will see copies of data accrued to date, or summaries of that data by treatment group. They will also assess the screening algorithm against the eligibility criteria. Emerging evidence from other related trials or research will be considered. Significant adverse events will be reviewed. The DSMC may tell the chair of the Trial Steering Committee at any time if, in their view, the trial should stop for ethical reasons. This includes concerns about participant safety. The DSMC will meet at least once per year during the recruitment phase of the study.

**Quality control**

The study may be monitored, or audited in accordance with the current approved protocol, relevant regulations and standard operating procedures by the Host organization, Sponsor or appropriate Regulatory Authorities. A Monitoring Plan will be developed according to Oxford Clinical Trials Research Unit standard operating procedures which involves a risk assessment. The monitoring activities are based on the outcome of the risk assessment and may involve central monitoring and site monitoring.

**Ethics and Dissemination**

Ethical approval was granted by Oxford C Research Ethics Committee, 28/04/2017, 17/SC/0207. Results of this study will be disseminated through peer reviewed publication and presentation at conferences.

**References**


1. The Royal College of Physicians. National Hip Fracture Database (NHFD) Annual Report 2017. London 2017.
2. The Royal College of Physicians. National Hip Fracture Database (NHFD) Annual Report 2015. London 2015.
3. Cooper C, Campion G, Melton LJ, 3rd. Hip fractures in the elderly: a world-wide projection. Osteoporos Int. 1992;2(6):285-9.
4. Johnell O, Kanis JA. An estimate of the worldwide prevalence, mortality and disability associated with hip fracture. Osteoporosis International. 2004;15(11):897-902.


For peer review only

5. Roche JJW, Wenn RT, Sahota O, Moran CG. Effect of comorbidities and postoperative complications on mortality after hip fracture in elderly people: prospective observational cohort study. BMJ. 2005;331(7529):1374.
6. Pollard TC, Newman JE, Barlow NJ, Price JD, Willett KM. Deep wound infection after proximal femoral fracture: consequences and costs. J Hosp Infect. 2006;63(2):133-9.
7. Theodorides AA, Pollard TC, Fishlock A, Mataliotakis GI, Kelley T, Thakar C, et al. Treatment of post-operative infections following proximal femoral fractures: our institutional experience. Injury. 2011;42 Suppl 5:S28-34.
8. Stannard JP, Volgas DA, Stewart R, McGwin Jr G, Alonso JE. Negative pressure wound therapy after severe open fractures: A prospective randomized study. Journal of Orthopaedic Trauma. 2009;23(8):552-7.
9. Shweiki D, Itin A, Soffer D, Keshet E. Vascular endothelial growth factor induced by hypoxia may mediate hypoxia-initiated angiogenesis. Nature. 1992;359(6398):843-5.
10. Saxena V, Hwang CW, Huang S, Eichbaum Q, Ingber D, Orgill DP. Vacuum-assisted closure: microdeformations of wounds and cell proliferation. Plast Reconstr Surg. 2004;114(5):1086-96; discussion 97-8.
11. Pietramaggiori G, Liu P, Scherer SS, Kaipainen A, Prsa MJ, Mayer H, et al. Tensile forces stimulate vascular remodeling and epidermal cell proliferation in living skin. Ann Surg. 2007;246(5):896-902.
12. Morykwas MJ, Argenta LC, Shelton-Brown EI, McGuirt W. Vacuum-assisted closure: a new method for wound control and treatment: animal studies and basic foundation. Ann Plast Surg. 1997;38(6):553-62.
13. Pollak AN. Use of negative pressure wound therapy with reticulated open cell foam for lower extremity trauma. J Orthop Trauma. 2008;22(10 Suppl):S142-5.
14. Webster J, Scuffham P, Stankiewicz M, Chaboyer WP. Negative pressure wound therapy for skin grafts and surgical wounds healing by primary intention. Cochrane Database Syst Rev. 2014;10:Cd009261.
15. England PH. Surveillance of surgical site infections in NHS hospitals in England: 2014 to 2015. 2015.
16. Noailles T, Brulefert K, Chalopin A, Longis PM, Gouin F. What are the risk factors for post-operative infection after hip hemiarthroplasty? Systematic review of literature. Int Orthop. 2015.
17. Edwards C, Counsell A, Boulton C, Moran CG. Early infection after hip fracture surgery: RISK FACTORS, COSTS AND OUTCOME. Journal of Bone & Joint Surgery, British Volume. 2008;90-B(6):770-7.
18. Wilson J, Charlett A, Leong G, McDougall C, Duckworth G. Rates of surgical site infection after hip replacement as a hospital performance indicator: analysis of data from the English mandatory surveillance system. Infect Control Hosp Epidemiol. 2008;29(3):219-26.
19. Singh S, Davies J, Sabou S, Shrivastava R, Reddy S. Challenges in reporting surgical site infections to the national surgical site infection surveillance and suggestions for improvement. Ann R Coll Surg Engl. 2015;97(6):460-5.
20. Tanner J, Padley W, Kiernan M, Leaper D, Norrie P, Baggott R. A benchmark too far: findings from a national survey of surgical site infection surveillance. J Hosp Infect. 2013;83(2):87-91.
21. Costa ML, Griffin XL, Achten J, Metcalfe D, Judge A, Pinedo-Villanueva R, et al. World Hip Trauma Evaluation (WHiTE): framework for embedded comprehensive cohort studies. BMJ Open. 2016;6(10).

For peer review only

22. NICE. The Management of Hip Fracture in Adults. 2014.
23. Centre for Disease Control. https://http://www.cdc.gov/nhsn/pdfs/pscmanual/9pscssicurrent.pdf 2018 [cited 2018 19/01/2018].
24. Haywood KL, Griffin XL, Achten J, Costa ML. Developing a core outcome set for hip fracture trials. Bone Joint J. 2014;96-b(8):1016-23.
25. Brooks R. EuroQol: the current state of play. Health Policy. 1996;37(1):53-72.
26. Oppe M, Devlin NJ, van Hout B, Krabbe PF, de Charro F. A program of methodological research to arrive at the new international EQ-5D-5L valuation protocol. Value Health. 2014;17(4):445-53.
27. Self-reports of health care utilisation: can a questionnaire replace a diary? 16th Annual meeting of the international society for health technology assessment in health care; 2000; The Hague, The Netherlands.

**Authors Contributions**

JM/MC/JA wrote the background section and developed the research question. JM/MC/JA/LS were responsible for the research methodology and management. JC wrote the sample size and statistical analysis sections of the protocol. MD wrote the health economic evaluation section of the protocol.

All authors reviewed and agreed the final manuscript.

**Competing interest**

No relevant competing interests to declare. ICMJE forms for each author held by corresponding author.

**Funding statement**

The study is funded by the Royal College of Surgeons of England and Dunhill Medical Trust and supported by the National Institute for Health Research (NIHR) Oxford Biomedical Research Centre. Excess treatment costs for the negative pressure wound therapy arm of the study are met by Smith and Nephew.

For peer review only

SPIRIT

STANDARD PROTOCOL ITEMS: RECOMMENDATIONS FOR INTERVENTIONAL TRIALS

SPIRIT 2013 Checklist: Recommended items to address in a clinical trial protocol and related documents*

| Section/item | Item No | Description | Addressed on page number |
|---|---|---|---|
| **Administrative information** | | | |
| Title | 1 | Descriptive title identifying the study design, population, interventions, and, if applicable, trial acronym | ________1______ |
| Trial registration | 2a | Trial identifier and registry name. If not yet registered, name of intended registry | _____2________ |
| | 2b | All items from the World Health Organization Trial Registration Data Set | _____2________ |
| Protocol version | 3 | Date and version identifier | _____1_______ |
| Funding | 4 | Sources and types of financial, material, and other support | _______15_______ |
| Roles and responsibilities | 5a | Names, affiliations, and roles of protocol contributors | ____1__________ |
| | 5b | Name and contact information for the trial sponsor | ____1_______ |
| | 5c | Role of study sponsor and funders, if any, in study design; collection, management, analysis, and interpretation of data; writing of the report; and the decision to submit the report for publication, including whether they will have ultimate authority over any of these activities | _____15________ |
| | 5d | Composition, roles, and responsibilities of the coordinating centre, steering committee, endpoint adjudication committee, data management team, and other individuals or groups overseeing the trial, if applicable (see Item 21a for data monitoring committee) | _____15______ |



For peer review only - http://bmjopen.bmj.com/site/about/guidelines.xhtml

For peer review only

| | | | |
|---|---|---|---|
| **Introduction** | | | |
| Background and rationale | 6a | Description of research question and justification for undertaking the trial, including summary of relevant studies (published and unpublished) examining benefits and harms for each intervention | __4________ |
| | 6b | Explanation for choice of comparators | ___5________ |
| Objectives | 7 | Specific objectives or hypotheses | ___5________ |
| Trial design | 8 | Description of trial design including type of trial (eg, parallel group, crossover, factorial, single group), allocation ratio, and framework (eg, superiority, equivalence, noninferiority, exploratory) | ___6_______ |
| **Methods: Participants, interventions, and outcomes** | | | |
| Study setting | 9 | Description of study settings (eg, community clinic, academic hospital) and list of countries where data will be collected. Reference to where list of study sites can be obtained | ___6________ |
| Eligibility criteria | 10 | Inclusion and exclusion criteria for participants. If applicable, eligibility criteria for study centres and individuals who will perform the interventions (eg, surgeons, psychotherapists) | _6-7_________ |
| Interventions | 11a | Interventions for each group with sufficient detail to allow replication, including how and when they will be administered | ___8________ |
| | 11b | Criteria for discontinuing or modifying allocated interventions for a given trial participant (eg, drug dose change in response to harms, participant request, or improving/worsening disease) | __12_______ |
| | 11c | Strategies to improve adherence to intervention protocols, and any procedures for monitoring adherence (eg, drug tablet return, laboratory tests) | ___N/A________ |
| | 11d | Relevant concomitant care and interventions that are permitted or prohibited during the trial | ___8________ |
| Outcomes | 12 | Primary, secondary, and other outcomes, including the specific measurement variable (eg, systolic blood pressure), analysis metric (eg, change from baseline, final value, time to event), method of aggregation (eg, median, proportion), and time point for each outcome. Explanation of the clinical relevance of chosen efficacy and harm outcomes is strongly recommended | ___8-10_____ |
| Participant timeline | 13 | Time schedule of enrolment, interventions (including any run-ins and washouts), assessments, and visits for participants. A schematic diagram is highly recommended (see Figure) | __10________ |



For peer review only - http://bmjopen.bmj.com/site/about/guidelines.xhtml

| | | | |
|---|---|---|---|
| Sample size | 14 | Estimated number of participants needed to achieve study objectives and how it was determined, including clinical and statistical assumptions supporting any sample size calculations | _____11____ |
| Recruitment | 15 | Strategies for achieving adequate participant enrolment to reach target sample size | ____11____ |

**Methods: Assignment of interventions (for controlled trials)**

| | | | |
|---|---|---|---|
| Allocation: | | | |
| Sequence generation | 16a | Method of generating the allocation sequence (eg, computer-generated random numbers), and list of any factors for stratification. To reduce predictability of a random sequence, details of any planned restriction (eg, blocking) should be provided in a separate document that is unavailable to those who enrol participants or assign interventions | ___7________ |
| Allocation concealment mechanism | 16b | Mechanism of implementing the allocation sequence (eg, central telephone; sequentially numbered, opaque, sealed envelopes), describing any steps to conceal the sequence until interventions are assigned | __7________ |
| Implementation | 16c | Who will generate the allocation sequence, who will enrol participants, and who will assign participants to interventions | __7________ |
| Blinding (masking) | 17a | Who will be blinded after assignment to interventions (eg, trial participants, care providers, outcome assessors, data analysts), and how | __7_________ |
| | 17b | If blinded, circumstances under which unblinding is permissible, and procedure for revealing a participant's allocated intervention during the trial | ____7_______ |

**Methods: Data collection, management, and analysis**

| | | | |
|---|---|---|---|
| Data collection methods | 18a | Plans for assessment and collection of outcome, baseline, and other trial data, including any related processes to promote data quality (eg, duplicate measurements, training of assessors) and a description of study instruments (eg, questionnaires, laboratory tests) along with their reliability and validity, if known. Reference to where data collection forms can be found, if not in the protocol | _10-11_______ |
| | 18b | Plans to promote participant retention and complete follow-up, including list of any outcome data to be collected for participants who discontinue or deviate from intervention protocols | __10-11_______ |



For peer review only - http://bmjopen.bmj.com/site/about/guidelines.xhtml

| | | | |
|---|---|---|---|
| Data management | 19 | Plans for data entry, coding, security, and storage, including any related processes to promote data quality (eg, double data entry; range checks for data values). Reference to where details of data management procedures can be found, if not in the protocol | ___11_______ |
| Statistical methods | 20a | Statistical methods for analysing primary and secondary outcomes. Reference to where other details of the statistical analysis plan can be found, if not in the protocol | __11-12____ |
| | 20b | Methods for any additional analyses (eg, subgroup and adjusted analyses) | ___11-12____ |
| | 20c | Definition of analysis population relating to protocol non-adherence (eg, as randomised analysis), and any statistical methods to handle missing data (eg, multiple imputation) | __11-12_____ |
| **Methods: Monitoring** | | | |
| Data monitoring | 21a | Composition of data monitoring committee (DMC); summary of its role and reporting structure; statement of whether it is independent from the sponsor and competing interests; and reference to where further details about its charter can be found, if not in the protocol. Alternatively, an explanation of why a DMC is not needed | ___12_____ |
| | 21b | Description of any interim analyses and stopping guidelines, including who will have access to these interim results and make the final decision to terminate the trial | ____12______ |
| Harms | 22 | Plans for collecting, assessing, reporting, and managing solicited and spontaneously reported adverse events and other unintended effects of trial interventions or trial conduct | _____10_____ |
| Auditing | 23 | Frequency and procedures for auditing trial conduct, if any, and whether the process will be independent from investigators and the sponsor | _13____ |
| **Ethics and dissemination** | | | |
| Research ethics approval | 24 | Plans for seeking research ethics committee/institutional review board (REC/IRB) approval | ___13_____ |
| Protocol amendments | 25 | Plans for communicating important protocol modifications (eg, changes to eligibility criteria, outcomes, analyses) to relevant parties (eg, investigators, REC/IRBs, trial participants, trial registries, journals, regulators) | __13_____ |



For peer review only - http://bmjopen.bmj.com/site/about/guidelines.xhtml

| | | | |
|---|---|---|---|
| Consent or assent | 26a | Who will obtain informed consent or assent from potential trial participants or authorised surrogates, and how (see Item 32) | ____7______ |
| | 26b | Additional consent provisions for collection and use of participant data and biological specimens in ancillary studies, if applicable | ____N/A______ |
| Confidentiality | 27 | How personal information about potential and enrolled participants will be collected, shared, and maintained in order to protect confidentiality before, during, and after the trial | ____11______ |
| Declaration of interests | 28 | Financial and other competing interests for principal investigators for the overall trial and each study site | Application |
| Access to data | 29 | Statement of who will have access to the final trial dataset, and disclosure of contractual agreements that limit such access for investigators | ___13______ |
| Ancillary and post-trial care | 30 | Provisions, if any, for ancillary and post-trial care, and for compensation to those who suffer harm from trial participation | __10______ |
| Dissemination policy | 31a | Plans for investigators and sponsor to communicate trial results to participants, healthcare professionals, the public, and other relevant groups (eg, via publication, reporting in results databases, or other data sharing arrangements), including any publication restrictions | __13_______ |
| | 31b | Authorship eligibility guidelines and any intended use of professional writers | _____N/A______ |
| | 31c | Plans, if any, for granting public access to the full protocol, participant-level dataset, and statistical code | ___13_______ |
| **Appendices** | | | |
| Informed consent materials | 32 | Model consent form and other related documentation given to participants and authorised surrogates | ____Supplementary files |
| Biological specimens | 33 | Plans for collection, laboratory evaluation, and storage of biological specimens for genetic or molecular analysis in the current trial and for future use in ancillary studies, if applicable | __N/A________ |

*It is strongly recommended that this checklist be read in conjunction with the SPIRIT 2013 Explanation & Elaboration for important clarification on the items. Amendments to the protocol should be tracked and dated. The SPIRIT checklist is copyrighted by the SPIRIT Group under the Creative Commons "Attribution-NonCommercial-NoDerivs 3.0 Unported" license.



For peer review only - http://bmjopen.bmj.com/site/about/guidelines.xhtml
